# Detection of the Malaria causing *Plasmodium* Parasite in Saliva from Infected Patients using Topoisomerase I Activity as a Biomarker

**DOI:** 10.1038/s41598-018-22378-7

**Published:** 2018-03-07

**Authors:** Marianne Smedegaard Hede, Søren Fjelstrup, Felix Lötsch, Rella Manego Zoleko, Anna Klicpera, Mirjam Groger, Johannes Mischlinger, Lilian Endame, Luzia Veletzky, Ronja Neher, Anne Katrine Wrist Simonsen, Eskild Petersen, Ghyslain Mombo-Ngoma, Magnus Stougaard, Yi-Ping Ho, Rodrigo Labouriau, Michael Ramharter, Birgitta Ruth Knudsen

**Affiliations:** 1 Zymonostics, Aarhus, Denmark; 2 Department of Molecular Biology and Genetics, University of Aarhus, Aarhus, Denmark; 3 Centre de Recherches Médicales de Lambaréné, Lambaréné, Gabon; 4 Department of Medicine, I, Division of Infectious Diseases and Tropical Medicine, Medical University of Vienna, Vienna, Austria; 5 Institut für Tropenmedizin, Universität Tübingen, Tübingen, Germany; 6 Department of Infectious Diseases, Aarhus University Hospital, Aarhus, Denmark; 7 Department of Infectious Diseases, The Royal Hospital, Muscat, Oman; 8 Department of Clinical Medicine, University of Aarhus, Aarhus, Denmark; 9 Division of Biomedical Engineering, Department of Electronic Engineering, The Chinese University of Hong Kong, Shatin, NT, Hong Kong SAR, China; 10 Department of Mathematics, University of Aarhus, Aarhus, Denmark

## Abstract

Malaria is among the major threats to global health with the main burden of disease being in rural areas of developing countries where accurate diagnosis based on non-invasive samples is in high demand. We here present a novel molecular assay for detection of malaria parasites based on technology that may be adapted for low-resource settings. Moreover, we demonstrate the exploitation of this assay for detection of malaria in saliva. The setup relies on pump-free microfluidics enabled extraction combined with a DNA sensor substrate that is converted to a single-stranded DNA circle specifically by topoisomerase I expressed by the malaria causing *Plasmodium* parasite. Subsequent rolling circle amplification of the generated DNA circle in the presence of biotin conjugated deoxynucleotides resulted in long tandem repeat products that was visualized colorimetrically upon binding of horse radish peroxidase (HRP) and addition of 3,3′,5,5′-Tetramethylbenzidine that was converted to a blue colored product by HRP. The assay was directly quantitative, specific for *Plasmodium* parasites, and allowed detection of *Plasmodium* infection in a single drop of saliva from 35 out of 35 infected individuals tested. The results could be determined directly by the naked eye and documented by quantifying the color intensity using a standard paper scanner.

## Introduction

Malaria is a widespread tropical disease causing approximately half a million deaths worldwide. The major burden of the disease is in rural areas of developing countries with limited access to modern clinics with sophisticated equipment and often with limited electricity supply^1^. Reliable diagnosis under these conditions remain a challenge. The emerging DNA sensors or sensor systems based on DNA for highly sensitive detection of biomarkers present attractive potential solutions to this challenge. Such developments are spurred by the recent advances in chemical synthesis of modified DNA oligonucleotides. As a result of such advances a large number of DNA sensors or sensor systems for the detection of different biomarkers including small molecules^2–5^, proteins^6,7^, or enzyme activities^7–15^ have been reported. Some of the DNA based sensors have been integrated with complex nano-structures^16–19^ while others have been used as stand-alone units^3,20^. Common for most sensors is that they are composed of relatively simple structures, exemplified by single- or double-stranded DNA structures containing Förster resonance energy transfer (FRET) pairs or DNA sequences that alternate between different DNA conformations upon external stimuli^3,20,21^.

DNA is an excellent material for highly sensitive detection systems due to the ease by which it can be amplified by easily accessible DNA polymerases^20,22,23^. One example of the amplification methods utilized in relation to DNA sensor systems is the rolling circle amplification (RCA) reaction catalyzed by e.g. phi29 polymerase or other polymerases with high levels of processivity combined with high strand-displacement activity^22^^,^^24,25^. As implied by the name, RCA generates multiple copies of circular DNA and has traditionally been used for the detection of specific nucleotide sequences^26–28^ that facilitate circularization of specific DNA molecules. More recently RCA-based techniques were also demonstrated successful for detection of small molecules^15,29–32^ or signals generated from enzymatic DNA cleavage-ligation reactions^12,33–39^. RCA is an isothermal reaction that utilizes a circular DNA template to generate long tandem repeat DNA products. These can be detected at the single–molecule level by a variety of visualization techniques, including cryo-transmission electron microscopy^40,41^, atomic force microscopy,^42,43^ or fluorescence microscopy after incorporation of fluorescent labeled nucleotides or hybridization with fluorescently labeled probes^15,34,36,44–46^. Hence, DNA sensor systems combined with RCA offer the dual advantages of being directly quantitative and highly sensitive, since each target molecule that generates a DNA circle results in a detectable product. When detecting enzyme activities the sensitivity is even further increased by the fact that each enzyme per definition can generate many DNA circle products, which can each be detected at the single molecule level, without being consumed in the process.

Combined with a specialized microfluidics based extraction system RCA enhanced enzyme activity detection (in short REEAD) was previously exploited for highly sensitive detection of the malaria causing *Plasmodium* parasites^34^. This was achieved using a DNA sensor module that was circularized specifically by the DNA cleavage-ligation activity of the *Plasmodium* specific enzyme, topoisomerase I (pTopI). Subsequently, the generated DNA circles where amplified by RCA and the products hybridized with fluorescent labeled probes before they where visualized at the single molecule level in a fluorescence microscope resulting in a detection limit of 0.06 parasites/μL using only a single drop of blood^34^. This sensitivity of pTopI specific REEAD was superior to the already developed gold standard diagnostic methods^47,48^ and better than most of the reported PCR protocols^49^ These findings suggest that the REEAD technology has the potential for being further developed into a highly sensitive diagnostic test. On top of that, the assay allowed detection of all *Plasmodium* species causing human malaria^34^. However, although the core of the assay, i.e. the pTopI mediated DNA circle generation and the subsequent RCA, did not require any thermal cycling or other sophisticated equipment the pump-driven microfluidics based extraction and microscopic readout both represented high technological and equipment heavy methods. Hence, for practical use in rural areas of developing countries where malaria is predominant, the previously described assay^34^ setup would be hampered by the limitations imposed by extraction and readout. These steps relied on electrical pumps and a fluorescence microscope, respectively. Circumventing such obstacles, we here report the successful replacement of i) the pump driven microfluidics device with a pump-free system, and ii) the microscopic readout with a direct visible colorimetric readout based on horse radish peroxidase (HRP) activity. These developments represent two important steps towards a novel point-of-care diagnostic method.

Due to the lifecycle of the malaria causing *Plasmodium* parasites, which involves a red blood cell stage, blood is the preferred sample type for diagnosis of malaria using gold standard thick- or thin smear microscopy^50^, PCR^49^ or rapid diagnostic tests (RDTs)^51,52^. However, we and others have reported the presence of trace amounts of *Plasmodium* derived nucleotide sequences^53–56^ and proteins^34,57,58^ in saliva from infected individuals although it is unlikely that viable *Plasmodium* parasites may be present in this specimen. In line with the high sensitivity of the described pTopI based assay we here demonstrate the successful detection of active *Plasmodium* enzymes in the saliva from 35 out of 35 malaria positive individuals. The possibility of using a noninvasive sample type such as saliva as test material for the pTopI based assay holds great promise for the future, since the REEAD technology, once fully developed, may be used for diagnosis in areas with cultural reluctance of giving blood as well as for eradication programs, which may involve testing of large number of asymptomatic individuals.

## Results

### Detection of *Plasmodium* infection using Saliva as the Test Material

The principle of the pTopI specific REEAD assay, which measures the activity of the essential *Plasmodium* expressed pTopI enzyme, and the combination of this assay with droplet microfluidic assisted sample extraction (REEAD-on-a-chip) has previously been described for specific and highly sensitive detection of *Plasmodium* parasites in blood samples from malaria positive individuals^34^. Furthermore, testing of a few saliva samples indicated the presence of active pTopI also in this sample type^34^.

**Figure 1:**
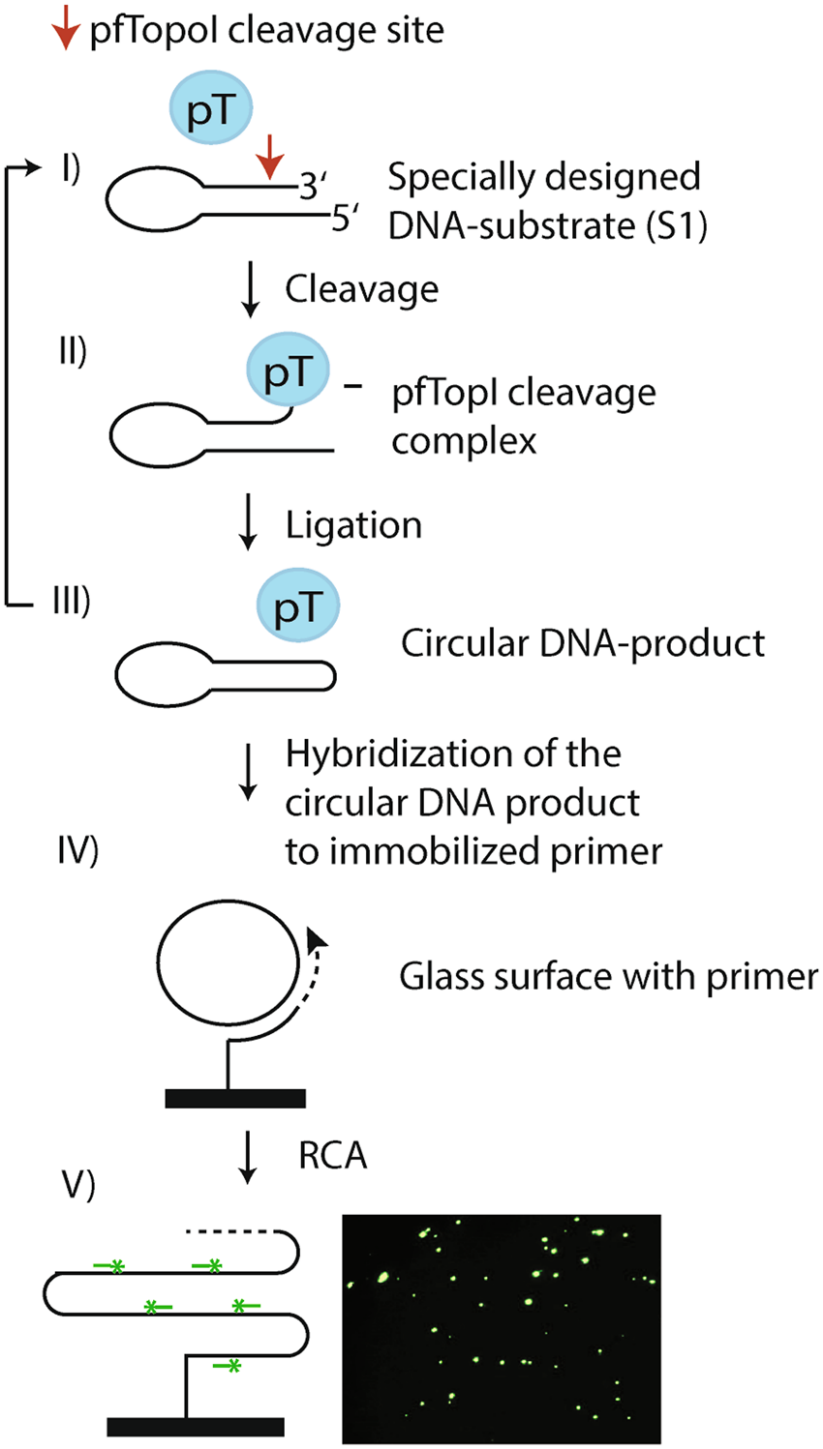
Schematic depiction of the REEAD assay. (**A**) I) The DNA-substrate (S1) carrying a pTopI cleavage site (red arrow). II) pTopI (represented by a blue oval labeled pT) mediated cleavage leads to covalent attachment of the enzyme to the DNA-substrate. This covalent intermediate is denoted the cleavage complex. In a subsequent pTopI mediated ligation step, III), a covalently closed DNA circle is formed and pTopI is released from the DNA and therefore able to catalyze a new cleavage/ligation event. IV) The circular DNA product is hybridized to an immobilized RCA primer, which is elongated in a RCA reaction. V) Left panel: The tandem repeat RCA product is detected using fluorescent probes (green) and fluorescence microscopy. V) Right panel: A typical microscopic view obtained performing the REEAD assay.

As illustrated in Fig. 1, the core of the REEAD assay is composed of a hairpin shaped DNA substrate, named S1 in the following (Table 1) that is converted to a closed DNA circle by the cleavage-ligation activity of pTopI. After hybridization to a specific primer the generated DNA circle functions as a template for a RCA reaction. This reaction generates a long tandem repeat product that can be visualized at the single molecule level by hybridization with fluorescently labeled probes with a nucleotide sequence complementary to a specific sequence on the RCA product. Subsequently, the individual RCA products, which each corresponds to a single cleavage-ligation reaction mediated by pTopI, can be visualized at the single molecule level under a fluorescence microscope. Consistent with the assay involving no thermal cycling it was demonstrated to be directly quantitative when measuring the number of signals obtained from serial diluted blood samples^34^.

**Table 1:** Overview of oligonucleotides used in the present study. All oligonucleotides were purchased at Sigma-Aldrich.

Oligonucleotide	Sequence
S1	TCTAGAAAGTATAGGAACTTCGAACGACTCAGAATGACTGTGAAGATCGCTTATCCTCAATGCACATGTTTGGCTCCCATTCTGAGTCGTTCGAAGTTCCTATACTTT
Amine-RCA primer	Amine-CCAACCAACCAACCAAATAAGCGATCTTCACAGT
RCA primer	CCAACCAACCAACCAAATAAGCGATCTTCACAGT
Detection probe	FAM-CCTCAATGCACATGTTTGGCTCC

The use of saliva as a test material for malaria diagnosis or detection of *Plasmodium* parasites in asymptomatic individuals will be highly valuable in areas with cultural reluctance of giving blood or for eradication programs. Therefore, in the current study we have addressed the potential of using pTopI specific REEAD for sensitive and specific detection of *Plasmodium* in saliva using a sample set of 52, including 23 frozen saliva samples from malaria positive individuals (Table 2, patient #1, #3–6, #8–25) and 29 frozen saliva samples from malaria negative individuals. The saliva samples collected from infected individuals were matched with corresponding blood samples taken from the same individuals at the same time. The blood samples were also tested by REEAD as well as by either RDT or, whenever possible, using standard thick smear microscopy, which allows estimation of the parasite concentrations. Information about the samples including their storage conditions and test results is summarized in Table 2. The negative samples were collected from uninfected individuals (including West Africans) residing in Denmark. Since it was relatively easy to obtain non-invasive samples it was possible to include more saliva (N = 29) than blood (N = 22) samples from uninfected individuals in the test. Matched saliva and blood samples from infected individuals were collected in Gabon. However, one of the blood samples was destroyed by coagulation and, therefore, 23 saliva and 22 blood samples from infected individuals were included in the test.

**Table 2:** Overview of samples from patients #1–#35. These samples were collected from malaria patients in Gabon during the time period June-December, 2014 (#1–#25), March 2016 (#26–#32) or February 2017 (#33–#35) and stored either frozen or at 4 °C as stated. Note that one patient sample (#32) was aliquoted and stored both at −20 °C and at 4 °C. The patients were diagnosed in Gabon using either thick smear microscopy or rapid diagnostic tests (RDT’s). The parasite concentrations in the patient blood samples, as determined by thick smear microscopy, are stated for samples #1–27. To reduce clotting of the microfluidic channels and enable reuse of the microfluidic chips, blood and saliva was diluted in PBS prior to microfluidics. Based on the parasite concentration in the blood sample the parasite concentrations in the diluted sample used for microfluidics was calculated. Due to different dilution factors this number is different for pump-driven microfluidics and hand-held microfluidics and both numbers are listed. Note that this dilution is not a technical prerequisite for analysis. In the current study, it was not possible to control the conditions (e.g. time and temperature) under which the samples were transported from the point of sampling to the point of analysis.* For saliva, the term “blood equivalent parasite concentration” is used to state the parasite concentration in the blood sample from the same patient if that blood sample was diluted with the same dilution factor as the saliva. ND: Not determined.

Patient no.	Parasite concentration in the collected blood sample (parasites/µL)	Parasite concentration (blood) or blood equivalent parasite concentration* (saliva) in inlet sample analyzed by pump driven microfluidics (parasites/µL)	Blood equivalent parasite concentration in inlet saliva samples analyzed by hand-driven microfluidics (parasites/µL)	Original diagnostic test	REEAD blood	REEAD saliva	Storage	Transfer conditions (time and temperature) from place of sampling to place of analysis
1	40	8	0.8	Thick smear	+	+	−20 °C	Unknown
2	80	16	1.6	Thick smear	ND	+	−20 °C	Unknown
3	200	40	4	Thick smear	+	+	−20 °C	Unknown
4	300	60	6	Thick smear	+	+	−20 °C	Unknown
5	360	72	7.2	Thick smear	+	+	−20 °C	Unknown
6	1200	240	24	Thick smear	ND	+	−20 °C	Unknown
7	1250	250	250	Thick smear	ND	+	−20 °C	Unknown
8	1300	260	26	Thick smear	+	+	−20 °C	Unknown
9	1550	310	31	Thick smear	+	+	−20 °C	Unknown
10	1650	330	33	Thick smear	+	+	−20 °C	Unknown
11	1750	350	35	Thick smear	+	+	−20 °C	Unknown
12	2500	500	50	Thick smear	+	+	−20 °C	Unknown
13	2800	560	56	Thick smear	+	+	−20 °C	Unknown
14	2900	580	58	Thick smear	+	+	−20 °C	Unknown
15	3300	660	66	Thick smear	+	+	−20 °C	Unknown
16	3600	720	72	Thick smear	+	+	−20 °C	Unknown
17	3800	760	76	Thick smear	+	+	−20 °C	Unknown
18	6300	1260	126	Thick smear	+	+	−20 °C	Unknown
19	7200	1440	144	Thick smear	+	+	−20 °C	Unknown
20	10700	2140	214	Thick smear	+	+	−20 °C	Unknown
21	18000	3600	360	Thick smear	+	+	−20 °C	Unknown
22	23900	4780	478	Thick smear	+	+	−20 °C	Unknown
23	38400	7680	768	Thick smear	+	+	−20 °C	Unknown
24	55000	11000	1100	Thick smear	+	+	−20 °C	Unknown
25	240000	48000	4800	Thick smear	+	+	−20 °C	Unknown
26	97400	19480	1948	Thick smear	ND	+	4 °C	Unknown
27	70700	14140	1414	Thick smear	ND	+	4 °C	Unknown
28	ND	ND	ND	RDT	ND	+	4 °C	Unknown
29	ND	ND	ND	RDT	ND	+	4 °C	Unknown
30	ND	ND	ND	RDT	ND	+	4 °C	Unknown
31	ND	ND	ND	RDT	ND	+	4 °C	Unknown
32	ND	ND	ND	RDT	ND	+	−20 °C /4 °C	Unknown
33	6900	1380	138	Thick smear	ND	+	4 °C	Unknown
34	2150	430	43	Thick smear	ND	+	−20 °C	Unknown
35	800	160	16	Thick smear	ND	+	−20 °C	Unknown

**Figure 2:**
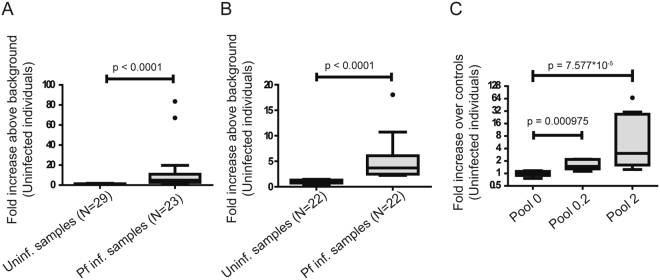
Detection of pTopI in patient samples. (**A**) Boxplot representing the results of testing 29 saliva samples from uninfected individuals and 23 samples from *Plasmodium* infected individuals (Table 2, patient #1, #3–6, #8–25) using the REEAD assay depicted in Fig. 1. (**B**) Boxplot representing the results of testing 22 blood samples from uninfected individuals and 22 samples from *Plasmodium* infected individuals (Table 2, #1, #3–5, #8–25) using the REEAD assay depicted in Fig. 1. (**C**) Boxplot representing the results of testing saliva diluted in such a way that blood from the same individual diluted in the same way would have a *Plasmodium* parasite concentration of 0.2 parasite/µL (Pool 0.2, N = 7); or 2 parasites/µL (Pool 2, N = 10). As a control saliva from 12 uninfected individuals was tested (Pool 0). NB: a base 2 logarithmic scale is used for the y-axis. In all cases the results are shown as fold increase above the average number of signals per microscopic frame obtained from analysis of samples from uninfected individuals.

All samples were tested using REEAD and the results analyzed in a fluorescence microscope essentially as described previously^34^. An X-Y scatter plot of the individual test results is shown in Supplementary Figure S1. Only sporadic signals were observed in the 29 saliva or 22 blood samples from malaria negative individuals. In comparison, testing of saliva or blood from malaria positive individuals in all cases resulted in signals that were at least two standard deviations above the mean of the signals obtained from analyses of malaria negative samples. The discriminatory power of the assay was highlighted by the fact that the medians of the results obtained from the group of samples from uninfected individuals were significantly different (p value < 0.0001) from that of the positive samples when analyzed using a Mann-Whitney U test regardless whether saliva (see box plot in Fig. 2A) or blood (box plot shown in Fig. 2B) was used as the test material. This result clearly demonstrates the ability of the REEAD assay to detect the presence of *Plasmodium* in saliva from malaria patients and verifies the presence of active pTopI in the saliva from infected individuals. Plotting the results obtained from each saliva sample as a function of parasite concentrations (measured in the matching blood samples), however, demonstrated that the number of REEAD signals observed did not correlate to the parasitemia of the tested individuals (Figure S1). The REEAD assay was directly quantitative when analyzing dilutions of the same samples (see previous published results^34^ and Fig. 2C). Hence, the observed lack of correlation between the number of REEAD signals and parasitemia when analyzing different saliva (or the matching blood) samples (Figure S1) may best be explained by the samples being collected at different times and treated differently in the time interval between sampling and testing by REEAD. Due to the long distance between sample collection and place of analyses in the current study, collection time and the precise storage conditions from collection to testing were very difficult to control and concerns in relation to these issues remain a subject for future investigations.

The detection limit of REEAD using blood as a test material was previously demonstrated to be as low as 0.06 parasites/µL^34^ simply by testing serial dilutions of blood with a known parasite concentration. Determination of the detection limit in saliva is more challenging since currently there is no standard method for quantification of parasite numbers in saliva samples. Therefore, only a rough estimation of the detection limit in saliva could be obtained. This was done by using the known parasite number in the matching blood samples as a reference for dilution of randomly picked saliva samples. Note, however, that this approach involved a number of uncertainties, not least due to the different quality of the available samples. In the experimental setup saliva samples from 7–10 individuals were each diluted with a factor equal to the dilution factor necessary to obtain a parasite concentration of 2 (referred to as pool 2 in the following) or 0.2 (referred to as pool 0.2 in the following) parasites/μL in the matching blood samples (termed blood equivalent parasite concentration (Table 2)) as described in materials and methods.

The results are shown as a box plot in Fig. 2C. Analyses of all samples (patients #1–3, #5, #7, #11, #14, #16, #18, #23; Table 2) (N = 10) in pool 2 resulted in a number of signals above the number of signals obtained when analyzing the uninfected control samples (Pool 0, N = 12) by at least 1.5 standard deviations. However, in pool 0.2 (patients #1, #2, #7, #14, #16, #18, #23; Table 2) (N = 7), one out of seven samples could not be distinguished from the pool 0 when analyzed by REEAD. A Kruskal-Wallis test shows that the three medians obtained when analyzing the three sample sets are not all equal (p-value = 2.6 × 10^−5^) and pairwise comparisons indicate that the medians of results obtained by analyzing pools 0 and 2, pools 0 and 0.2, and pools 0.2 and 2 are all statistically different with p-values of 7.577 × 10^−5^, 0.0009753 and 0.03179, respectively. These results suggest that *Plasmodium* infections may be detected in saliva samples even from patients with a relatively low parasite concentration of 2 parasites/μL in the blood stream.

### REEAD enabled *Plasmodium* detection based on unfrozen saliva samples

For long term storage of field collected samples and to ensure experimental reproducibility, all saliva samples tested hitherto were frozen in aliquots prior to testing. For the potential future use of the REEAD assay as a diagnostic tool the method will most probably be used to test unfrozen saliva samples. Therefore, to investigate if freezing is a necessary step to achieve efficient extraction of pTopI from saliva and obtain positive REEAD results, fresh saliva samples were collected in Gabon and transported at 4 °C to Europe where they were tested by REEAD within 5 to 45 days post collection.

**Figure 3:**
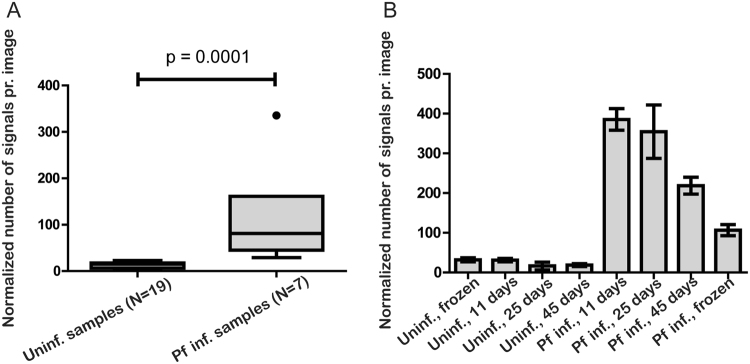
Detection of pTopI in unfrozen saliva from malaria patients: (**A**) Boxplot showing the results from the REEAD assay for testing unfrozen saliva from uninfected individuals (N = 19) or *Plasmodium* infected persons (N = 7) (#26–32, Table 2). (**B**) Bar chart showing the results of analyzing a saliva sample that had either been frozen upon arrival or kept at 4 °C for 11, 25, or 45 days. Due to limited amount of sample it was not possible to do repetitions of the analysis of the positive samples. The error bars on the bars representing the results of testing the positive samples therefore show the standard error of measurement for 16 randomly selected microscopic images. All results were normalized to the results obtained using a sample from patient #12 (Table 2) as reference sample.

Immediately upon arrival of the samples (corresponding to five to six days post collection), saliva from seven malaria positive (#26–32, Table 2) and 19 uninfected individuals was analyzed using the REEAD assay. The box plot in Fig. 3A shows the average number of signals per image frame obtained from 16 images. To avoid noise arising from slide to slide variation all results were normalized to the results obtained using a sample from patient #12 (Table 2) as reference sample. The signals obtained from *Plasmodium* positive samples were in all cases above the mean of the signals arising from malaria negative samples with at least 2 standard deviations (data not shown), clearly demonstrating the ability of REEAD to detect malaria using unfrozen saliva as the test material. In concert with this finding, a Mann-Whitney U test shows that the median of the results obtained from the group of samples from uninfected individuals differ significantly from the median of the results obtained from the group of samples from *Plasmodium* infected individuals (P = 0.0001).

Malaria is prevalent in areas with unstable electricity and, hence, without stable freezing capacity. In order to test the vulnerability of REEAD to storage of saliva samples outside a freezer, aliquots from patient #32 (see Table 2) were stored at 4 °C and tested by REEAD 11, 25, and 45 days after sample acquisition in comparison to 3 samples from malaria negative individuals stored under similar conditions. Moreover, aliquots of the sample from patient #32 (Table 2) were frozen five days after the time of collection and analyzed in comparison to the unfrozen aliquots. The results from the frozen aliquots were used for normalization to compensate for slide-to-slide variations.

As evident from the bar chart in Fig. 3B, the number of signals obtained when analyzing the unfrozen sample did not decrease relative to the starting point (five days post collection) for the first 25 days of storage, while after 45 days the number of signals declined with a factor ∼2. After 45 days of storage at 4 °C analyses of the unfrozen samples still resulted in approximately double amount of signals relative to the frozen aliquot of a sample taken from the same patient. These results demonstrate that freezing is not a necessary step for sample extraction and that unfrozen saliva may in fact be a more suited sample for REEAD analysis compared to the frozen saliva used for the analyses shown in Fig. 2.

### Establishment of a colorimetric readout for REEAD based *Plasmodium* detection

**Figure 4:**
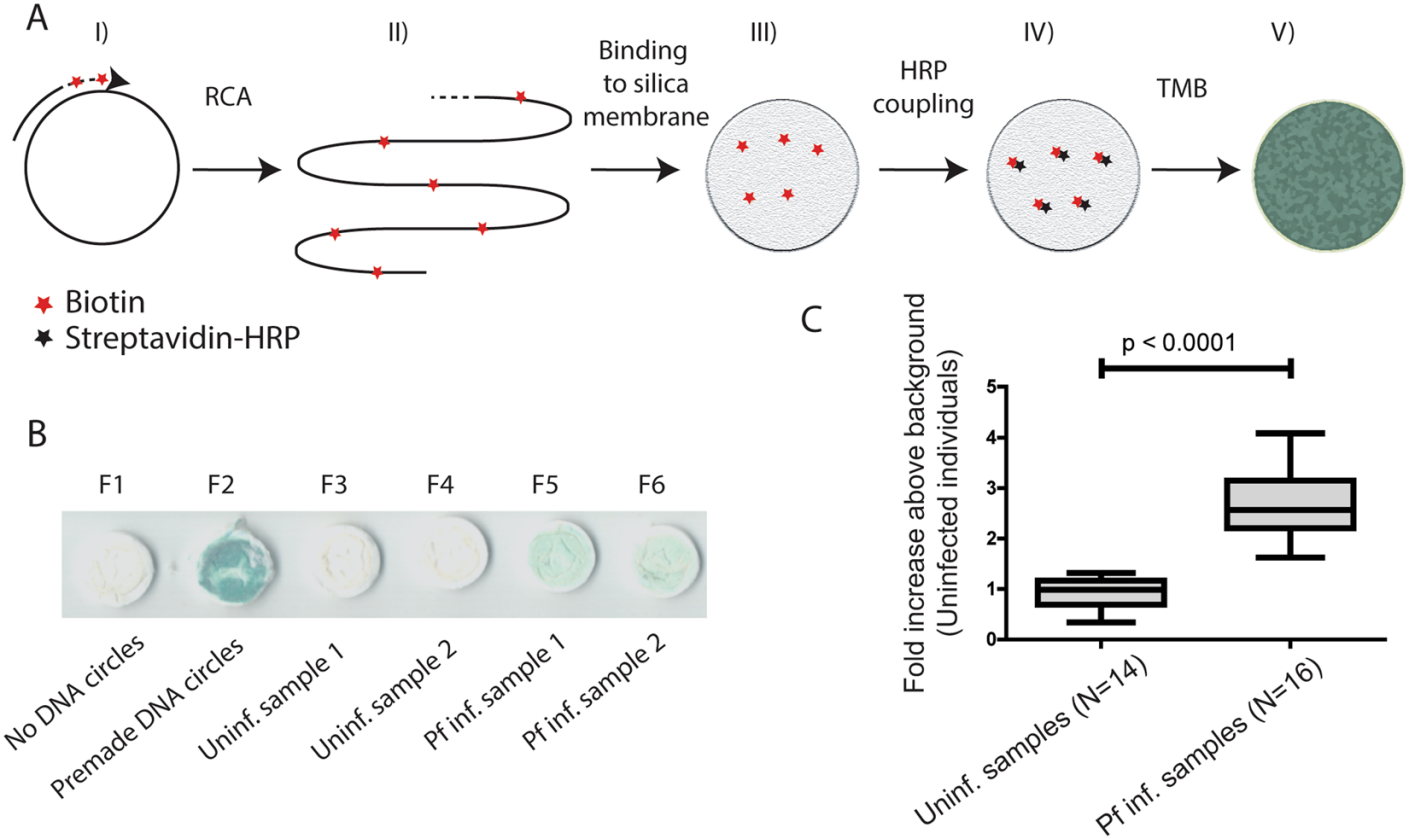
Detection of pTopI using a colorimetric readout. (**A**) The individual steps of the colorimetric readout are schematically depicted. (I) shows the RCA primer hybridized to the pTopI generated single stranded DNA circles. II) The primer is elongated in a RCA reaction performed in the presence of biotin labeled nucleotides (red asterisks). III) The biotinylated DNA generated is bound to a silica membrane, which is positioned in a column. IV) Silica bound biotinylated DNA is visualized by coupling to streptavidin conjugated HRP (black asterisks) followed by V) incubation with the colorimetric HRP substrate, TMB. (**B**) Representative pictures of the silica membranes after completion of the colorimetric readout using either a sample without DNA circles or spiked with premade DNA circles (F1 and F2 respectively) as well as after testing saliva samples from uninfected individuals (F3 and F4) or *Plasmodium* infected individuals (F5 and F6) (patients #4 and #20 respectively, see Table 2). (**C**) Box plot representing the result of using the above described colorimetric readout for testing 14 saliva samples from uninfected individuals and 16 samples from *Plasmodium* infected individuals. The results are shown as fold increase above the average signal obtained from the analysis of samples from uninfected individuals.

The experiments described above were all performed using a fluorescence microscope to detect the REEAD signals. Such method is not suited for the low-resource areas where malaria is prevalent. To allow visualization of the RCA products resulting from pTopI activity, using a protocol that is more adaptable to the settings in low-income countries, a colorimetric readout was developed. In this setup, which is schematically depicted in Fig. 4A, the RCA reaction of the pTopI generated DNA circles is performed in solution in the presence of biotin conjugated deoxynucleotides, which are incorporated into the RCA products. The biotinylated RCA products are then bound to a silica filter and visualized through coupling to streptavidin-HRP followed by addition of the colorimetric HRP substrate, 3,3′,5,5′-Tetramethylbenzidine (TMB), directly to the silica membranes. A representative picture of the results obtained using this readout is shown in Fig. 4B. To the left are shown the results obtained when analyzing a negative sample containing no DNA circles (F1) and a positive control sample containing 0.1 pmol of premade DNA circles (F2), respectively. F3 and F4 show the results of analyzing two saliva samples from uninfected individuals. F5 and F6 are typical examples of the results obtained when analyzing saliva from malaria patients (#4 and #20, Table 2).

The colorimetric REEAD setup as exemplified in Fig. 4B, was tested using 16 frozen saliva samples from 12 different malaria patients (#1 (tested twice), #2, #4 (tested 3 times), #7, #11, #13, #16, #18, #20 (tested twice), #21–23 (tested once); see Table 2 for parasite concentrations) and 14 frozen samples from uninfected individuals. Note, that only some of the samples could be tested in repetition due to the limited sample volumes. The colorimetric signals generated when analyzing each of the samples were documented by scanning the silica filters using a paper scanner and quantified using the Image J software. The results are shown as a box plot in Fig. 4C. All malaria positive samples gave rise to signal intensities at least 2 standard deviations above the mean signal intensities obtained when analyzing malaria negative samples. Moreover, a Mann-Whitney U test shows that the medians of the results obtained from the samples taken from uninfected persons differ significantly from the median of the results obtained from analyzing samples from *Plasmodium* infected persons (P < 0.0001). Taken together these results strongly suggest that the malaria specific REEAD setup can be combined with a simple yet efficient colorimetric readout, which is a crucial step towards adapting the REEAD technology to low resource settings.

### Development of a pump-free system for extraction of pTopI from saliva samples

**Figure 5:**
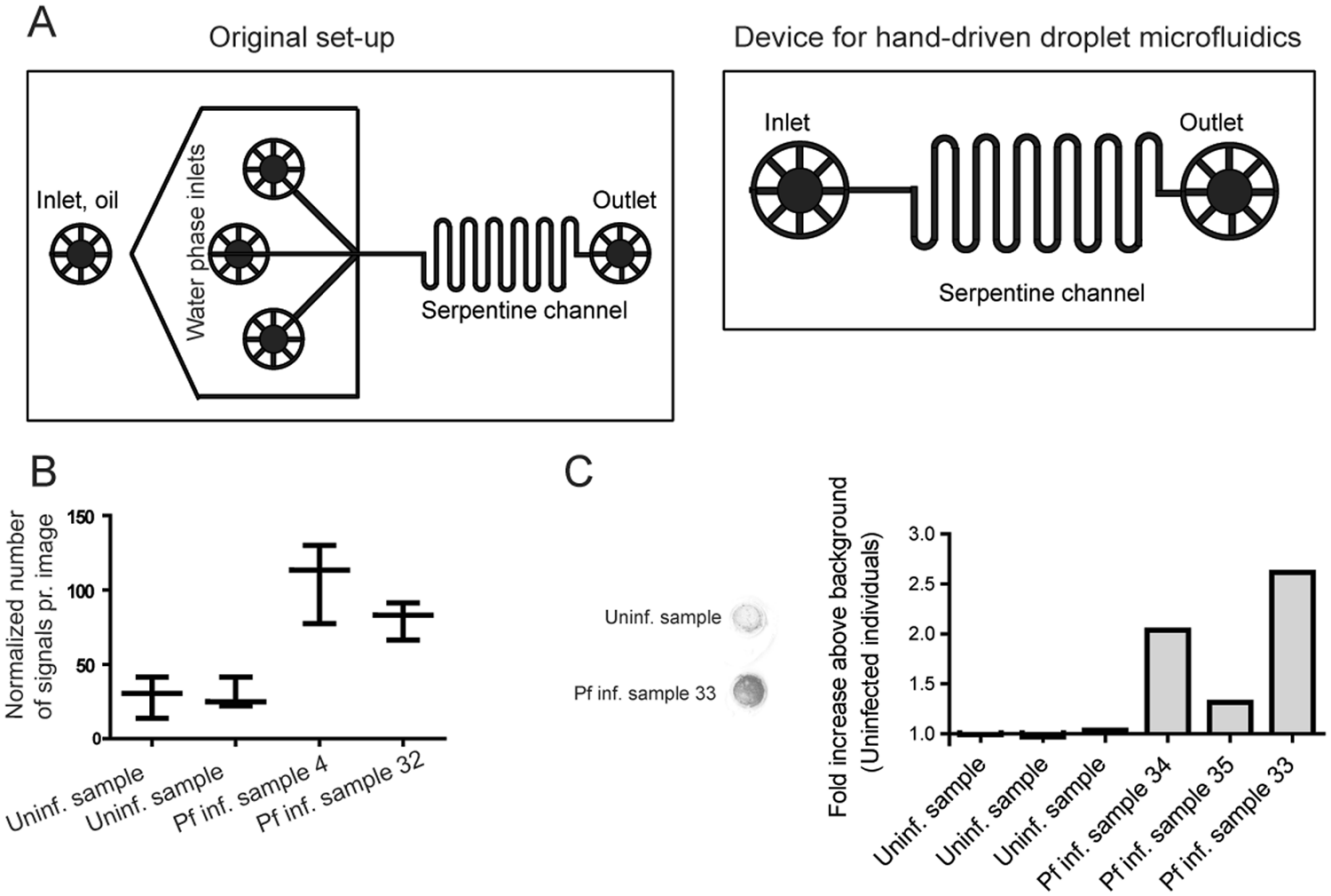
Detection of pTopI using a pump-free extraction method. (**A**) Schematic illustration of the microfluidic device used for pump-driven (top panel) and pump-free (lower panel) extraction of pTopI from saliva from *Plasmodium* infected individuals. In the case of pump-driven droplet microfluidics, patient sample, S1, and lysis buffer are fed into the microfluidic chip through the 3 inlets. By competition with oil (fed into the system through a 4^th^ inlet) pL sized water in-oil-droplets are generated. The droplets are then led through a serpentine channel and collected. In the case of pump-free extraction, water-in-oil droplets are made by vortexing saliva, lysis buffer, S1, and oil. The droplets are then loaded into the microfluidic device using a handheld syringe and collected from the outlet after passage through a serpentine channel. (**B**) Extraction of saliva from uninfected or *Plasmodium* infected individuals was done using the pump-free microfluidics method depicted in A). The boxplot represents the results of testing two samples from uninfected persons and two samples from persons with malaria (patients #4 and #32, see Table 2). The results shown represent the results of three independent extractions of each sample. The results were normalized to the results obtained using a sample from patient #12 (Table 2) as reference sample (**C**) Left panel; representative image of the results obtained when combining pump-free microfluidics with the colorimetric read-out described in Fig. 4. The figure shows a filter after completion of the assay using a sample from an uninfected individual and a sample from a *plasmodium* infected individual (patient #33, see Table 2). Right panel; quantitative depiction of the result obtained when testing 6 samples using the pump-free extraction combined with colorimetric readout. The results are shown as fold-increase over the average of the readings obtained when testing negative samples. Hence, the negative samples give numbers varying around 1.

In the tests described above, pTopI from patient samples were extracted using a so-called water-in-oil droplet microfluidics device. In this device (see Fig. 5A, top panel) the sample to be analyzed, the REEAD substrate (S1), and the hypotonic lysis buffer was loaded into individual channels of the microfluidic device. When these three aqueous streams merged they were broken up by an oil stream to form stable water-in-oil emulsions. Each aqueous drop served as a micro-reactor, which subsequently was pushed through a serpentine channel to ensure adequate mixing of reagents. As demonstrated previously the enhanced reaction kinetics observed in the picoliter droplets ensured efficient detection using even very small (a single drop) unprocessed samples for analysis. A drawback of the standard setup described above is that it involves pump driven control of the flow-rates of both the oil and the aqueous phases, which is unsuited for low resource settings.

To circumvent the need of electrical pumps a pump-free extraction procedure was developed. This was achieved by generating the water-in-oil droplets by vortexing oil with a mixture of sample, substrate S1, and hypotonic lysis buffer. Though the droplets produced in this manner are heterogeneous, the confined droplets still offer the enhanced reaction kinetics necessary for effective extraction and substrate conversion^34^, when manually loaded in to a simplified microfluidic chip composed of a single inlet and outlet interrupted by a serpentine channel (Fig. 5A, lower panel). Droplets generated using this pump-free method were analyzed by REEAD following the same protocol as the droplets generated by the pump driven system and readout was based on microscopic visualization as described. Two saliva samples from uninfected individuals were analyzed together with two saliva samples from *Plasmodium* infected persons (Table 2, patients #4 and #32). The results of three independent extractions of each sample are shown in the box plot in Fig. 5B. As evident from the plot, analysis of the malaria positive samples resulted in signals above the mean of the signals obtained from the two malaria negative samples by at least 2 standard deviations. This result indicates that a handheld extraction method may replace the current pump driven method. Moreover, it was possible to combine the simplified hand driven extraction and mixing protocol with the above described colorimetric readout (Fig. 5C) holding promise for the future adaptation of the described method for a new malaria diagnostic tool based on saliva as the sample type and suitable for use in low-income countries. Note, that the analyses shown in Fig. 5C was only performed once due to low sample volume.

## Conclusion

We here demonstrate the functionality of a REEAD protocol specific for the activity of the *Plasmodium* expressed enzyme pTopI for sensitive detection of malaria causing parasites using saliva from infected individuals as the test material. The documentation of saliva as a usable sample type for REEAD based detection of *Plasmodium* infections demonstrates the presence of active pTopI in this specimen. We found that although the REEAD method in principle is quantitative in nature the number of signals obtained when analyzing saliva samples from patients with different parasitemias did not correspond to the parasite concentrations in the blood. Since testing was done far from the sample collection in both space and time the lack of correlation between the number of REEAD signals and parasite load in tested individuals may reflect differences in storage conditions and time from collection to testing. Indeed, signals were observed to decline with storage of infected saliva over time even when the samples where kept at a steady temperature of 4 °C. Potential differences in storage conditions of the samples available for this study also impose a degree of uncertainty on the estimated detection limit (estimated to a blood equivalent parasite concentration of 2 parasites/μL) in frozen saliva. For a more precise determination of the detection limit and to address if the observed lack of correlation between REEAD signal and parasite load could be ascribed to sample quality, testing of larger number of freshly collected saliva samples close to the site of collection is being planned but is out of the scope of the current study. Indeed, such study will require the establishment of a test kit suitable for low resource settings. In the current manuscript, we present the development and testing of the individual components of such kit i.e. a pump free microfluidics based extraction of pTopI and a colorimetric readout allowing detection directly by the naked eye. Moreover, holding promise for future development of an all-in-one REEAD based kit for field testing of malaria in saliva specimen we demonstrate the feasibility of combining the pump free extraction with colorimetric readout for detection of malaria in laboratory settings. Even if future field tests should prove that saliva cannot allow quantitative testing of malaria, we believe that the use of non-invasive sample type for the detection of *Plasmodium* infections will continue to be appealing not least for eradication programs, which may involve tests of large number of asymptomatic individuals.

## Materials and Methods

### Oligonucleotides

#### Blood and saliva samples from malaria patients and uninfected individuals

Blood samples from individuals that are not infected with malaria were obtained from the blood bank at the Aarhus University Hospital, Skejby. Matching blood and saliva samples from patients diagnosed with malaria were obtained at the CERMEL, Albert Schweitzer Hospital, Lambaréné, Gabon. The patients were diagnosed using thick smear microscopy or by RDT’s (see Table 2). The study was conducted in accordance to the Declaration of Helsinki, and samples were collected and analyzed following local regulations and guidelines. Ethical clearance (#003/2014) for sampling in Gabon was obtained from the institutional ethics committee at the Centre de Recherches Médicales de Lambaréné. Informed consent was obtained from all study participants or their legal guardians in case of minors. Thick smear microscopy was performed by two independent readers. A third reading was performed if required (e. g. if first and second reading differed more than 50% of parasite count or if the same slide was read positive/negative by different readers). The number of counted parasites per µL blood were recorded. The final parasitemia was determined as the average of first and second reading (or, if third reading was performed: the average of the two closest results). In the case of testing by RDTs, the “Paracheck Pf” test was used.

Note that samples were diluted in PBS prior to microfluidics in order to avoid clotting of the microfluidic chip. This is not a prerequisite for the analyses but reduced the number of microfluidic chips necessary to conduct the analyses in this laboratory test.

#### Pump Driven Droplet Microfluidics

Unless otherwise stated in the result section pTopI was extracted from blood or saliva from malaria patients using pump driven droplet microfluidics. The droplet microfluidic devices (see Fig. 5A for design) were fabricated by conventional soft lithography techniques^59^, casting and curing the PDMS prepolymer on a SU-8 3025 (MicroChem) master of a channel height at around 25 μm. PDMS prepolymer (Sylgard 184) was prepared in a 10:1 (base:curing agent) ratio and cured at 65 °C for 1 hour. Prior to the experiments, the channels were wetted with oil/surfactant (Pico-Surf 1, 2% in HFE-7500, Dolomite Microfluidics) for at least 15 minutes. Two syringe pumps (Harvard Apparatus) were used to control the flow rates of oil/surfactant and reagents independently. The droplet volume and generation frequency were controlled by the flow rate ratio, determined by the competition between continuous phase and disperse phase^34,60,61^. Blood/saliva (diluted 1:4 in PBS), S1 substrate (Table 1 and^12^ − 167 nM final concentration in droplets) and lysis buffer (10 mM Tris pH 7.5; 5 mM EDTA; 0.2% Tween 20; 1 mM DTT; 1 mM PMSF) were subjected to droplet microfluidics essentially as described previously^34^. The procedure was completed by breaking the droplets by addition of 25%(v/v) 1 H,1 H,2 H,2H-Perfluoro-1-octanol (Sigma-Aldrich).

#### Pump-free Droplet Microfluidics

For pump-free droplet microfluidics (used in Fig. 5) a slightly modified microfluidic device design (see Fig. 5A) was used. This design consists of a serpentine channel flanked by a single inlet and a single outlet. Saliva was diluted 50 times in PBS and mixed with lysis buffer (10 mM Tris pH 7.5; 5 mM EDTA; 0.2% Tween 20; 1 mM DTT; 1 mM PMSF), the DNA substrate S1 (167 nM) (Table 1 and^12^), and Pico-Surf 1–2% in HFE-7500 in the same ratios as described previously^34^. Water-in-oil droplets were produced by vortexing for 1 minute. Using a hand-held syringe, the hereby generated droplets were subsequently fed into the single channel microfluidic chip. Following collection of the droplets from the outlet the droplets were broken by addition of 25% (v/v) 1 H,1 H,2 H,2H-Perfluoro-1-octanol.

#### pTopI assay

An amine coupled oligo (Amine-RCA primer, see Table 1) was immobilized on NHS-coated microscopy slides (CodeLink**®** HD Activated Slides) as described by the supplier (Surmodics).

Lysate supplemented with NaCl to a final concentration of 500 mM was added to the DNA conjugated slides to allow hybridization of pTopI generated DNA circles. Subsequent phi29 DNA polymerase enabled rolling circle amplification (RCA) and hybridization of a fluorescent detection probe (Table 1) to the RCA products was performed as described previously^34^. Fluorescence labeled RCA products were visualized in a fluorescence microscope (Olympus IX73) and quantified by counting the number of signals per microscopic image frame using the ImageJ software. The results were obtained by counting the number of pTopI specific signals on 16 randomly picked microscopic pictures (277,3 × 234 µm^2^) for each sample analyzed. Inherent to this readout is some slide to slide variation due to variations in coupling efficiency of the surface attached primer^36^. Therefore, to allow comparison across experiments/slides the results are shown as fold increase over the average of the number of signals achieved from the negative samples tested at the same time.

Generally, the data are presented as a box and whiskers plot with Tukey-style whiskers made using the GraphPad Prism software.

#### Colorimetric readout

The RCA primer was elongated by phi29 DNA polymerase (Life technologies) in the presence of 20 V% lysate (see Pump-driven droplet microfluidics), 10 µM RCA primer (Table 1) and 0.1 mM of each dNTP of which, 10% of the dCTP has been replaced by Biotin-16-Aminoallyl-2′-dCTP (TriLink Biotechnologies). Following primer elongation, the RCA product was diluted 10 fold in TE buffer (10 mM Tris-HCl, pH 7.5; 1 mM EDTA), and then 4 fold using CP buffer from E.Z.N.A. Cycle Pure Kit (OMEGA bio-tek). The diluted RCA products were bound to a DNA purification column (OMEGA bio-tek, Catalog number: D6492–02) and allowed to bind for 10 minutes on a shaker at room temperature. The entire reaction volume was then spun through the column (approximately 10 seconds in a table top centrifuge). Following binding of the RCA products, the column was washed with 200 µL PBS (pH 7.5) and blocked with 200 µL blocking buffer (5% skimmed milk in 100 mM Tris-HCl, pH 7.5) for 10 minutes. The column was then washed twice with 200 µL PBS, incubated with 200 µL 8 M Urea for 10 minutes, and washed with 200 µL PBS. 150 µL Streptavidin-HRP (Pierce Streptavidin Poly-HRP, Life Technologies) in blocking buffer (8 µL/mL blocking buffer) was then added to the column, which was allowed to incubate for 10 minutes. The column was then washed 3 times with 150 µL PBS supplemented with 0.2% Triton X-100. Finally, 150 µL TMB Enhanced HRP Membrane Substrate (ESPM) (Surmodics) was added to the columns. The reaction was allowed to run for 10 minutes after which the columns were spun at 10000 g for 10 seconds and the reaction stopped using 150 µL 1 M sulphuric acid. Finally, the columns were washed with 150 µL ddH2O.

The filters were removed from the columns and scanned using a HP Scanjet 5590 P paper scanner. The scanned image was split into the three RGB channels, and the red channel was analyzed using ImageJ. The “measure” function at standard settings was used to quantify the amount of reacted TMB. A standard value of 30.000, corresponding to an uncolored filter, was subtracted from all samples. The results are presented as fold increase over the average of the signal intensity achieved from the negative samples tested at the same time.

## Electronic supplementary material

Supplementary information
