# Supplementary material for: Detection of the Malaria causing Plasmodium Parasite in Saliva from Infected Patients using Topoisomerase I Activity as a Biomarker
Source: Sci Rep. 2018 Mar 7;8:4122. doi: 10.1038/s41598-018-22378-7 (PMC5841400; doi:10.1038/s41598-018-22378-7)
Supplement: Supplementary file 1 — Supplementary information [file 41598_2018_22378_MOESM1_ESM.doc]

**Supplementary Information**

**Detection of the Malaria causing *Plasmodium* Parasite in Saliva from Infected Patients using Topoisomerase I Activity as a Biomarker**

Marianne Smedegaard Hede^a^, Søren Fjelstrup^b^, Felix Lötsch^c,d^, Rella Manego Zoleko^c^, Anna Klicpera^c^, Mirjam Groger^c^, Johannes Mischlinger^c,d,e^, Lilian Endame^c^, Luzia Veletzky^c^, Ronja Neher^c,e^, Anne Katrine Wrist Simonsen^b^, Eskild Petersen^f,g^, Ghyslain Mombo-Ngoma^c,e^, Magnus Stougaard^h^, Yi-Ping Ho^i^, Rodrigo Labouriau^j^, Michael Ramharter^c,d,e^, Birgitta Ruth Knudsen^b^.

^a^ Zymonostics, Aarhus, Denmark.

^b^ Department of Molecular Biology and Genetics, University of Aarhus, Denmark.

^c^ Centre de Recherches Médicales de Lambaréné, Lambaréné, Gabon.

^d^ Department of Medicine, I, Division of Infectious Diseases and Tropical Medicine, Medical University of Vienna, Austria.

^e^ Institut für Tropenmedizin, Universität Tübingen, Tübingen, Germany.

^f^ Department of Infectious Diseases, Aarhus University Hospital, Aarhus, Denmark.

^g^ Department of Infectious Diseases, The Royal Hospital, Muscat, Oman.

^h^ Department of Clinical Medicine, University of Aarhus, Denmark.

^i^ Division of Biomedical Engineering, Department of Electronic Engineering, The Chinese University of Hong Kong, Shatin, NT, Hong Kong SAR, China.

^j^ Department of Mathematics, University of Aarhus, Denmark.

The ability of the REEAD-assay described in figure 1 to discriminate between samples obtained from uninfected people and samples obtained from *plasmodium* infected people was tested. The performance of the assay was tested using either saliva (Figure S1, A) or blood samples (Figure S1, B) as test material.


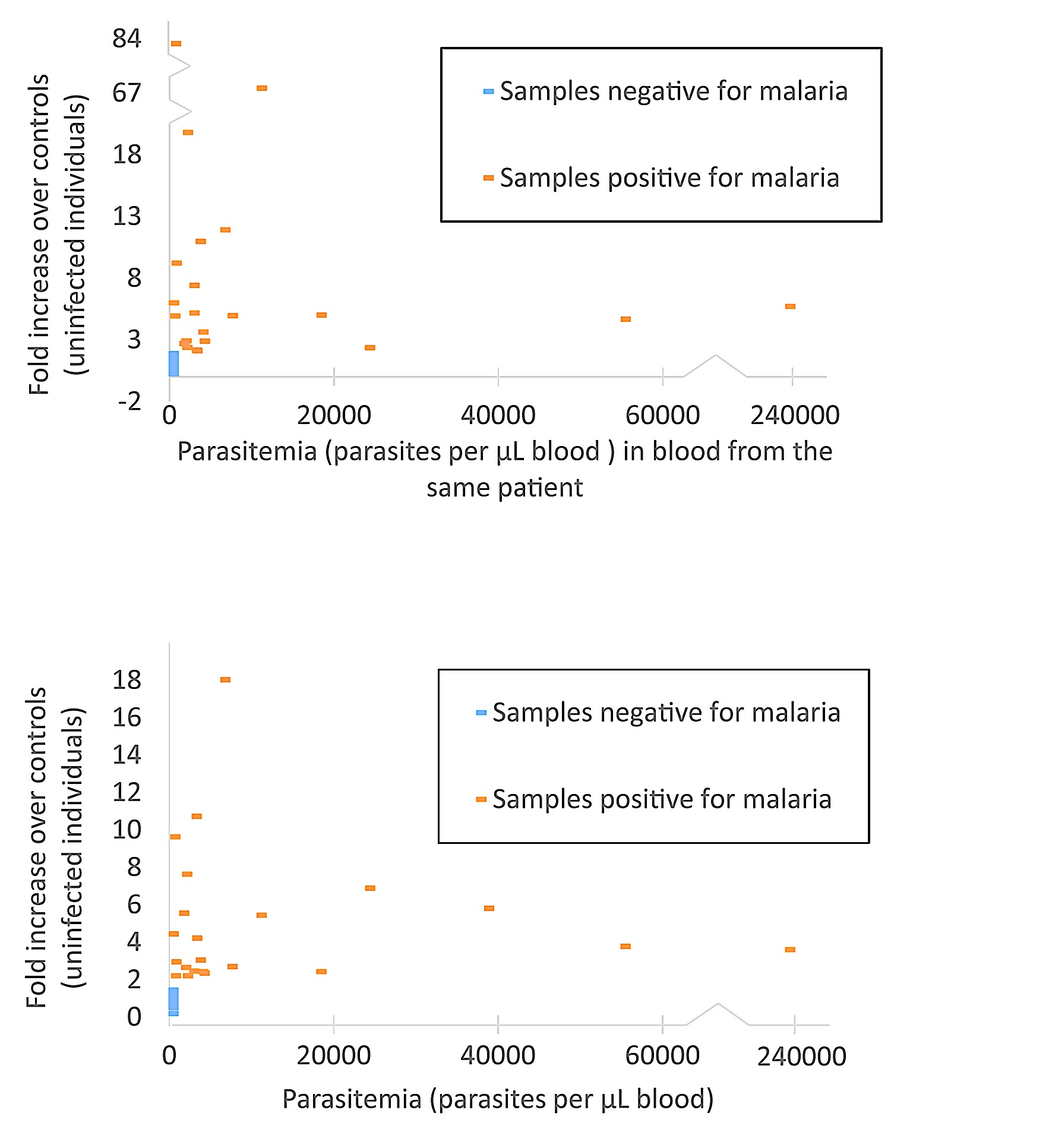
Supplementary figure S1

A)

B)

Supplementary figure S1. A, shows the results of testing 29 saliva samples from uninfected individuals and 23 saliva samples from *Plasmodium* infected individuals (table 2, patient #1, #3-6, #8-25). B, shows the results obtained from testing 22 blood samples from uninfected individuals and 22 samples from *Plasmodium* infected individuals (table 2, #1, #3-5, #8-25) using the REEAD assay. The number of fluorescent signals per image frame was determined and the fold increase over controls was calculated. The results are shown as a XY-scatter plot correlating parasitemia (X-axis) and signal strength (Y-axis). B.

Blue line: samples negative for malaria. Red line: samples positive for malaria
